# Explanation

**Published:** 1896-02

**Authors:** 


### EXPLANATION.

WE regret that, through an error in the make-up of the January number, the resolutions of the Boston Society for Dental Improvement failed to accompany the picture of Professor Chandler. In justice to that organization, the picture is repeated and placed in its proper connection with the resolutions.

